# Establishment of a loop-mediated isothermal amplification-lateral flow dipstick assay for the point-of-care testing of feline herpesvirus-1

**DOI:** 10.3389/fvets.2026.1863672

**Published:** 2026-07-06

**Authors:** Shushuai Yi, Han Zhao, Jing Huang, Yuwei Cai, Jing Wu, Wei Lian, Jiangting Niu, Baishuang Yin

**Affiliations:** 1 College of Animal Science and Technology, Jilin Agricultural Science and Technology College, Jilin, China; 2 Jilin Zhengye Biological Products Co., Ltd., Jilin, China

**Keywords:** feline herpesvirus-1, LAMP-LFD, point-of-care testing, TK gene, visual detection

## Abstract

**Introduction:**

Feline herpesvirus-1 (FHV-1) is a major pathogen responsible for feline upper respiratory tract disease (URTD), with a global distribution that poses significant threats to both the health of domestic cat and the conservation of wild felids. Thus, the development of a rapid and specific diagnostic method is crucial for controlling FHV-1 infection. In this study, we established a visual detection assay for FHV-1 by combining loop-mediated isothermal amplification (LAMP) with lateral flow dipstick (LFD).

**Methods:**

The assay utilized six of primers targeting the highly conserved TK gene of FHV-1. The LAMP amplification was performed using biotin-labeled H-FIP and 6-FAM-labeled H-LF, followed by visual detection using LFD. After optimizing the reaction conditions, the LAMP-LFD assay was established and systematically evaluated for its specificity, sensitivity, and repeatability. Furthermore, the performance of this assay was assessed using nasal swabs from 87 cats suspected with feline URTD.

**Results:**

The optimal reaction conditions were determined as follows: 0.2 μM each of outer primers, 1.2 μM each of inner primers, and 0.6 μM each of loop primers, with amplification at 63 °C for 40 min. The assay showed no cross-reactivity with other pathogens caused feline URTD, demonstrating high specificity. The detection limit was 10^2^ copies/μL for recombinant plasmid and 10^1.5^ TCID_50_/mL for FHV-1 viral culture. Good repeatability was also confirmed. Clinical testing of 87 nasal swabs revealed positive rates of 49.43% (43/87). Compared to qPCR, the assay exhibited a specificity of 97.56%, sensitivity of 91.30%, and an overall agreement of 94.25%, with a kappa value of 0.89, indicating almost perfect concordance between the two methods.

**Discussion:**

The LAMP-LFD assay developed in this study is highly sensitive, specific, simple, independent of specialized equipment and easy to perform with results visualized by the naked eye, making it well-suited for point-of-care testing of FHV-1 in primary veterinary hospitals and field setting.

## 1 Introduction

Feline viral rhinotracheitis (FVR), caused by feline herpesvirus-1 (FHV-1), is a highly contagious upper respiratory tract disease (URTD) in cats, characterized primarily by coughing, sneezing, serous or purulent ocular and nasal discharges, conjunctivitis, keratitis, and ulcers around the eyes (1). FHV-1 is an enveloped, linear double-stranded DNA virus classified within the genus *Varicellovirus*, subfamily *Alphaherpesvirinae*, family *Herpesviridae* (2). Virion of FHV-1, measuring approximately 120–180 nm in diameter, consists of genomic DNA, capsid, tegument and envelope. The FHV-1 genome is approximately 136 kb in length, encoding 23 virus-associated proteins and 13 glycoproteins (3). Among these, gB, gC, gD, gG, gH, and gL play critical roles in regulation of viral replication and infection, thymidine kinase (TK), gE, gI, and PK are closely associated with viral pathogenicity and neurotropism (4). FHV-1 primarily infects cats via the nasal, oral, and conjunctival routes. Kittens between 2 and 4 months are most susceptible to FHV-1, as maternal antibodies decline by 8 weeks after birth. Upon infection, approximately 80% of cats develop latent infection, with the virus persisting in the trigeminal and optic ganglia (5). Among these, about 45% of latently infected cats may shed virus spontaneously or in response to natural stressors, thereby serving as key reservoirs for transmission (1, 5, 6). Viral latency and recurrent shedding represent major challenges for the effective control and prevention of FHV-1 infection.

Currently, both inactivated and attenuated live vaccines are available for the prevention of FHV-1, while antiviral drugs such as famciclovir, ganciclovir, and interferon are used for treatment following infection (7, 8). Despite these measures, FHV-1 continues to exhibit high prevalence and incidence rates among domestic cats worldwide. The virus remains particularly widespread in high-density environments such as animal shelters, stray cat populations, and multi-cat households (9). Clinical signs caused by FHV-1 present similar to those of other feline upper respiratory pathogens, such as feline calicivirus (FCV), *Mycoplasma felis* (*M. felis*), *Chlamydia felis* (*C. felis*), and *Bordetella bronchiseptica* (*Bb*), making differential diagnosis based solely on clinical signs highly challenging (10). Moreover, FHV-1 exhibits a broad host range among felids except domestic cats, including European wildcats (11), snow leopard (12), cheetahs (13), mountain lions (14), Siberian tigers (15) and South China tiger (16), which poses a significant threat to the conservation of wild felids, particularly those that are already vulnerable or endangered. Consequently, it is imperative to develop rapid, specific, and sensitive point-of-care testing (POCT) methods for FHV-1 detection in clinical and field setting.

To facilitate efficient diagnosis of FHV-1 infection, a variety of molecular detection methods have been developed and popularized, including PCR-based assays (PCR, nested PCR, nanoPCR, and qPCR) (17–19), recombinase polymerase amplification (RPA)-based methods (exo-RPA, RPA-lateral flow dipstick (RPA-LFD) and RAA-CRISPR/Cas12a-LFS) (20–23). However, PCR-based assays require a thermal cycler for target gene amplification, limiting its use in primary animal hospital with limited infrastructure or field settings. RPA-based assay enables rapid amplification without sophisticated equipment, while the extremely fast reaction kinetics of RPA is difficult to control, and its high cost poses additional challenges for widespread adoption. Loop-mediated isothermal amplification (LAMP) utilizes the strand displacement activity of *Bst* DNA polymerase and 4–6 specifically primers to efficiently amplify target genes under isothermal conditions (24). LAMP offers strong specificity, high sensitivity, low cost, and simple operation, making it widely applicable in pathogen detection. Notably, the integration of LAMP with LFD enables visual readout via test lines, offering significant potential for POCT and holding considerable promise for clinical applications (25).

In this study, we developed a visual LAMP-LFD assay targeting the TK gene for rapid detection of FHV-1 and evaluated its specificity and sensitivity, providing a cost-effective diagnostic tool suitable for point-of-care testing of FHV-1 in primary animal hospital and field setting.

## 2 Materials and methods

### 2.1 Plasmid, virus and extraction of viral DNA

One plasmid pMD-TK, containing the TK gene of FHV-1, was constructed in our previous study and used as a positive template for the development of LAMP-LFD assay in this study. FHV-1 CH-B was isolate from the nasal discharge of domestic cat suspected of FVR, and propagated in Crandell-Rees Feline Kidney (CRFK) cells. The viral titer reached 10^7.5^ TCID_50_/mL. FCV CH-JL2, feline panleukopenia virus (FPV) CC-02/16, *Bb* YN16-1 and *Streptococcus pneumoniae* (*Sp*) YN22-7 were all isolated and maintained in our laboratory. *C. felis*-, *M. felis*- and Canine herpesvirus 1 (CHV-1)-positive nasal discharges and feline coronavirus (FCoV)-positive ascites were confirmed through qPCR and sequencing analysis, and kept in our laboratory. Total nucleic acids were extracted following the manufacturer’s instructions of FastPure Viral DNA/RNA Mini Kit (Vazyme, China). The concentrations of the extracted nucleic acids and plasmid pMD-TK were measured using a NanoDrop spectrophotometer (Thermo Scientific, United States), and the gene copies were calculated using the following equation: Copy number (copies/μL) = [6.02 × 10^23^ × concentration of DNA × 10^−9^ (ng/μL)]/(entire length of DNA × 650).

### 2.2 Design of primers and probes

**Figure 1:**
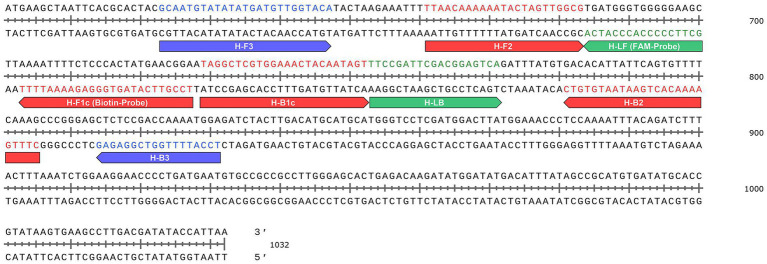
Binding sites of LAMP-LFD primers targeting TK gene of FHV-1 isolate C-27 (Genbank accession number: FJ478159). The sequences marked with blue, red, and green represent two outer primers (H-F3 and H-B3), two inner primers [H-FIP (F1c-F2) and H-BIP (B1c-B2)], and two loop primers (H-LF and H-LB), respectively. Primer FIP was labeled with biotin at the 5′ end, and primer H-LF was labeled with 6-FAM at the 5′ end, with amplification in the 5′-3′ direction.

TK gene of FHV-1 is highly conserved, exhibiting approximately 100% nucleotide identity among different isolates, and has been widely used as a target gene in the development of molecular detection assays for FHV-1. In this study, TK gene was also determined as the target gene to design primer set of LAMP assay using the software Primer explorer version 5.^1^ Specificity, dimer and hairpin formations of primers were analyzed using online software Primer Blast and MFEprimer.^2^ The primer set consisted of two outer primers (H-F3 and H-B3), two inner primers (H-FIP and H-BIP), and two loop primers (H-LF and H-LB), of which primer H-FIP was labeled with biotin at the 5′ end, and the primer H-LF was labeled with 6-Carboxyfluorescein (6-FAM) at the 5′ end. Figure 1 shows the locations of the primers within the TK gene, and Table 1 lists the oligonucleotide sequences of all primers. All primers were synthesized by Comate Bioscience Co., Ltd. (Jilin, China).

**Table 1:** Oligonucleotide sequences of primers used for LAMP-LFD.

Primer name	Primer sequences (5′-3′)
H-F3	GCAATGTATATATGATGTTGGTACA
H-B3	TCCATTTTGGTCGGAGAG
H-FIP	Biotin-TCCGTTCATAGTGGGAGAAAATTTTTTAACAAAAAATACTAGTTGGCG
H-BIP	TAGGCTCGTGGAAACTACAATAGTCTTTGAAAACACTGAATAATGTGTC
H-LF	6-FAM-GCTTCCCCCACCCATCA
H-LB	TTCCGATTCGACGGAGTCA

### 2.3 LAMP reaction system

The LAMP reaction was performed using WarmStart^®^ Multi-Purpose LAMP/RT-LAMP 2 × Master Mix (New England Biolabs, United States). The reaction system was 25 μL, which contained 12.5 μL of WarmStart Multi-Purpose LAMP/RT-LAMP 2 × Master Mix, 2.5 μL of 10 × primer mix (2 μM each of H-F3 and H-B3, 16 μM each of H-FIP and H-BIP, and 4 μM each of H-LF and H-LB), 2.0 μL of template DNA and 8.0 μL of nuclease-free water. Plasmid pMD-TK was used as positive control, and nuclease-free water as negative control. The LAMP amplification was carried out using a metal bath at 65 °C for 60 min, and then terminated reaction at 80 °C for 5 min. LAMP amplicons were suitable for visual detection via LFD strips, as well as agarose gels electrophoresis analysis (showed in Supplementary Figures).

### 2.4 Optimization of the FHV-1 LAMP reaction system

To determine the optimal reaction conditions of FHV-1 LAMP assay, real-time fluorescent LAMP reactions using EvaGreen as the fluorescent dye were performed using the LAMP reaction system described in Section “2.3 LAMP reaction system,” with the addition of 1 × EvaGreen dye to the reaction mixture. Amplification was carried out on a LightCycler^®^ 96 instrument (Roche, Switzerland) with the following parameters: incubation at the set constant temperature for 60 min, and fluorescence signals were collected every 45 s. The following parameters were systematically optimized as single variables. Amplification temperatures were tested at 58 °C to 67 °C with 1 °C gradient interval. Inner primers were evaluated at a final concentration of 0.2, 0.4, 0.6, 0.8, 1.0, 1.2, 1.4, 1.6, and 1.8 μM. Loop primers were assessed at a final concentration of 0.2, 0.4, 0.6, 0.8, 1.0, 1.2, 1.4 and 1.6 μM. The optimal reaction parameters were determined by analyzing the amplification curves in combination with the time-to-positivity (Tp) values and endpoint fluorescence intensities. Furthermore, the amplification products were also used for visual detection with LFD strips. To eliminate errors, all reactions were performed in triplicate.

### 2.5 Establishment of the FHV-1 LAMP-LFD assay

**Figure 2:**
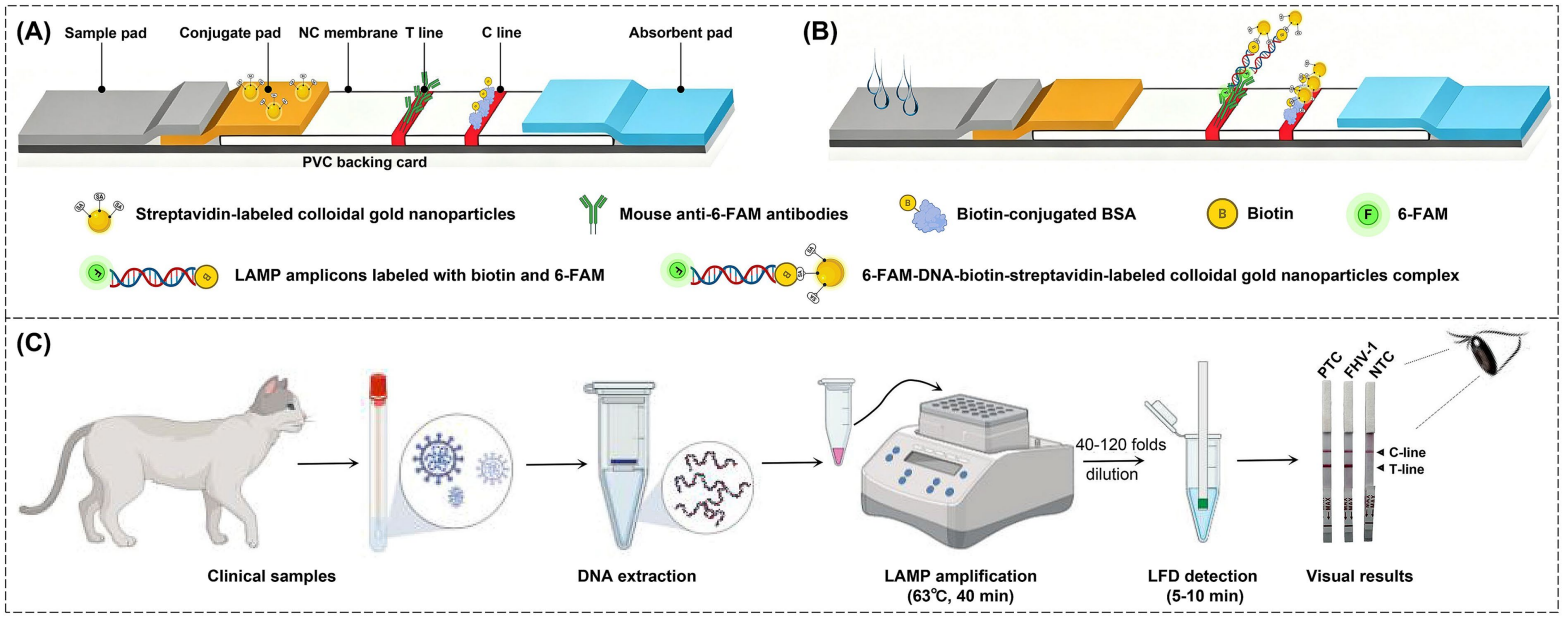
Schematic illustration of the detection of FHV-1 by LAMP-LFD. **(A)** Design schematic of the fundamental configurations of the LFD. **(B)** Diagram of the detection principle of LAMP-LFD. **(C)** Schematic diagram of the testing procedure for FHV-1 LAMP-LFD assay.

Following LAMP amplification under the optimal reaction system, the commercial LFD strips (GenDx, China) were used for the detection of the resultant amplicons. As shown in Figure 2A, the LFD strip consists of sample pad, conjugate pad, nitrocellulose (NC) membrane, absorbent pad, and polyvinyl chloride (PVC) backing card. Colloidal gold nanoparticles (20–40 nm) labeled with streptavidin were sprayed onto the conjugate pad. On the NC membrane, mouse anti-6-FAM antibodies (1 mg/mL) and biotin-conjugated bovine serum albumin (BSA) (1 mg/mL) were sprayed onto the test line (T-line) and control line (C-line) using a film dispenser, respectively. After drying at 37 °C for 12 h, the sample pad, conjugate pad, NC membrane, and absorbent pad were sequentially laminated onto the PVC backing card for strip assembly. Briefly, the NC membrane was fixed in the center of the PVC backing card, with the sample pad and absorbent pad attached to its two ends, respectively. The conjugate pad was placed between the NC membrane and the sample pad, with an overlap of 1–2 mm at each junction. The assembled strips were cut into 3 mm-wide bands and stored under dry conditions. The FHV-1 LAMP assay was performed using primer H-FIP labeled with biotin and primer H-LF labeled with 6-FAM, yielding a large amount of stem-loop structure DNA products labeled with biotin and 6-FAM. The LAMP products were mixed with nuclease-free water and then applied to the sample pad of LFD strip. The products specifically bound to the streptavidin-labeled colloidal gold nanoparticles on the conjugate pad, forming a ternary complex (6-FAM-DNA-biotin-streptavidin-labeled colloidal gold nanoparticles). Subsequently, these complexes migrated toward the absorbent pad via capillary action and specifically bound to the anti-6-FAM antibody on the T-line, resulting in the aggregation of colloidal gold nanoparticles and the formation of a visible red band. Excess streptavidin-labeled colloidal gold nanoparticles continued to migrate and bound to the biotin-conjugated BSA on the C-line, forming a second visible red band (Figure 2B). Therefore, the presence of only the C-line indicated a negative result, the appearance of both the C-line and T-line indicated a positive result, while the absence of the C-line rendered the test invalid (Table 2).

**Table 2:** Comparative analysis of LAMP-LFD and qPCR for the detection of FHV-1 in clinical nasal swabs.

LAMP-LFD	Reference assay (qPCR)			Specificity (%) (95% CI)	Sensitivity (%) (95% CI)	*k* value^*^	Agreement (%)
	Positive	Negative	Total				
Positive	42	1	43	97.56 (92.83–100.00)	91.30 (83.16–99.45)	0.89	94.25
Negative	4	40	44				
Total	46	41	87				

*Based on the kappa (*k*) values, a value of less than 0.20 indicated a poor agreement, 0.21–0.40 indicated a fair agreement, 0.41–0.60 indicated a moderate agreement, 0.61–0.80 indicated a substantial agreement, 0.81–1.00 indicated a almost perfect agreement.

### 2.6 Optimization of the reaction time of LAMP-LFD assay

To determine the optimal reaction time, LAMP was performed under the optimized reaction system using plasmid pMD-TK (10^4^ copies/μL) and nuclease-free water as templates, respectively. The reactions were incubated in a metal bath at 63 °C for different times ranging from 10 min to 60 min with 10 min gradient interval. Following the manufacturer’s instructions for the commercial LFD strips, LAMP amplicons were subjected to a 40–120 fold dilution using nuclease-free water. LFD strip was vertically inserted into the diluted solution for 5–10 min, and the results were subsequently interpreted.

### 2.7 Assessment of the specificity, sensitivity and repeatability of the FHV-1 LAMP-LFD assay

To determine the specificity, the nucleic acids of FHV-1, FCV, FPV, *C. felis*, *M. felis*, *Bb*, *Sp*, FCoV and CHV-1 were used as templates for LAMP-LFD detection.

To assess the sensitivity, 10-fold serial dilutions of pMD-TK plasmid ranging from 10^0^ copy/μL to 10^7^ copies/μL were tested using LAMP-LFD assay. Viral DNA extracted from 10-fold serial dilutions of FHV-1 culture at concentrations ranging from 10^0.5^ to 10^7.5^ TCID_50_/mL were also used to determine the limit of detection (LOD) of the LAMP-LFD assay. Furthermore, the LOD of the LAMP-LFD for both plasmid and FHV-1 culture was compared with that of a commercialized qPCR test kit for FHV-1.

To evaluate the repeatability, three diluted plasmids with the concentration of 10^2^ copies/μL, 10^4^ copies/μL and 10^6^ copies/μL were tested using the LAMP-LFD assay. The experiment repeated three times for each concentration.

### 2.8 Application of the LAMP-LFD assay for POCT of FHV-1

A total of 87 nasal swabs, which collected from suspected FVR cases from four animal hospitals in Jilin province, China, were tested using LAMP-LFD and commercialized qPCR kit. The coincident rate, comparative sensitivity, comparative specificity, and kappa coefficient value were calculated using two-by-two table based on qPCR as reference assay.

## 3 Results

### 3.1 Optimization of LAMP reaction conditions

**Figure 3:**
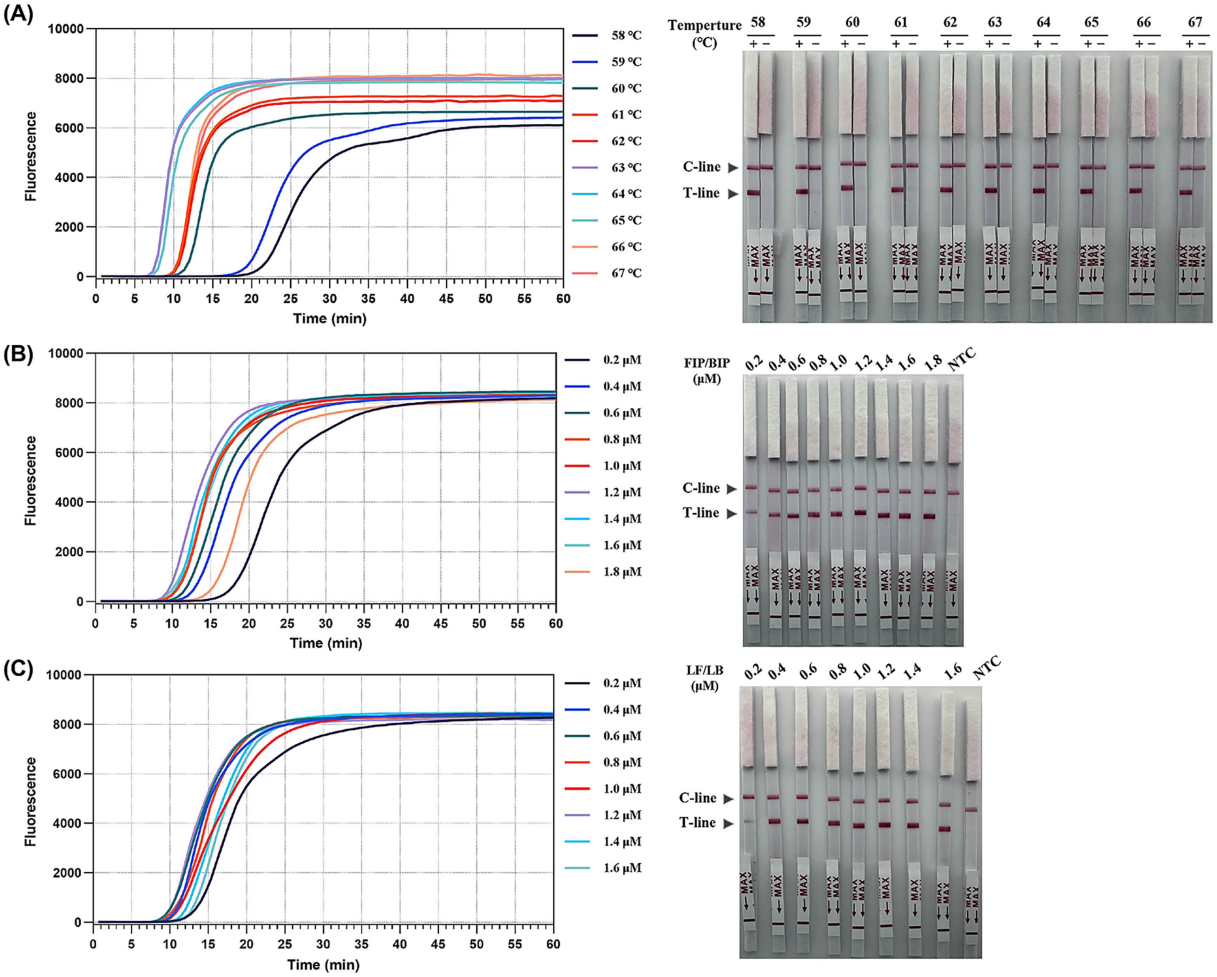
Optimization of the LAMP reaction system for the detection of FHV-1. Using 10^6^ copies/μL of plasmid pMD-TK as a template to optimized amplification temperature **(A)**, the inner primers concentration **(B)**, and the loop primers concentration **(C)**. The left panel shows the amplification curves of real-time fluorescence LAMP, and the right panel presents the visual detection results of the LFD. “+” represent positive reaction with plasmid pMD-TK as a template; “−” and NTC represent negative controls.

The amplification temperature, and primers concentration for the FHV-1 LAMP assay were optimized using the single control variable method. A real-time fluorescent LAMP system was used to optimize the reaction conditions based on the fluorescence amplification curve. The reaction temperature was optimized first. The results showed that specific fluorescence amplification curves were generated in all reactions using plasmid pMD-TK as template after incubation at 58–67 °C for 60 min, and all reactions completed exponential amplification and entered the plateau phase within 35 min. Furthermore, LFD strip detection revealed distinct T-lines in the positive controls at all tested reaction temperatures, whereas only C-lines were observed in the negative controls. According to the LAMP fluorescence amplification curves, the lowest Tp value and the highest endpoint fluorescence intensity were obtained at 63 °C, corresponding to the highest amplification efficiency (Figure 3A). Therefore, 63 °C was determined as the optimal reaction temperature for FHV-1 LAMP assay.

Subsequently, the concentrations of the inner primers and loop primers were optimized sequentially. As shown in Figures 3B,C, the highest amplification efficiencies were achieved at 1.2 μM for inner primer and 0.6 μM for loop primer, respectively. Consistent with the fluorescence results, LFD analysis confirmed that these concentrations yielded more intense coloration at the T line. Therefore, the optimal final concentrations for outer, inner and loop primers were determined as 0.2 μM, 1.2 μM and 0.6 μM.

### 3.2 Optimization of the reaction time of LAMP-LFD assay

**Figure 4:**
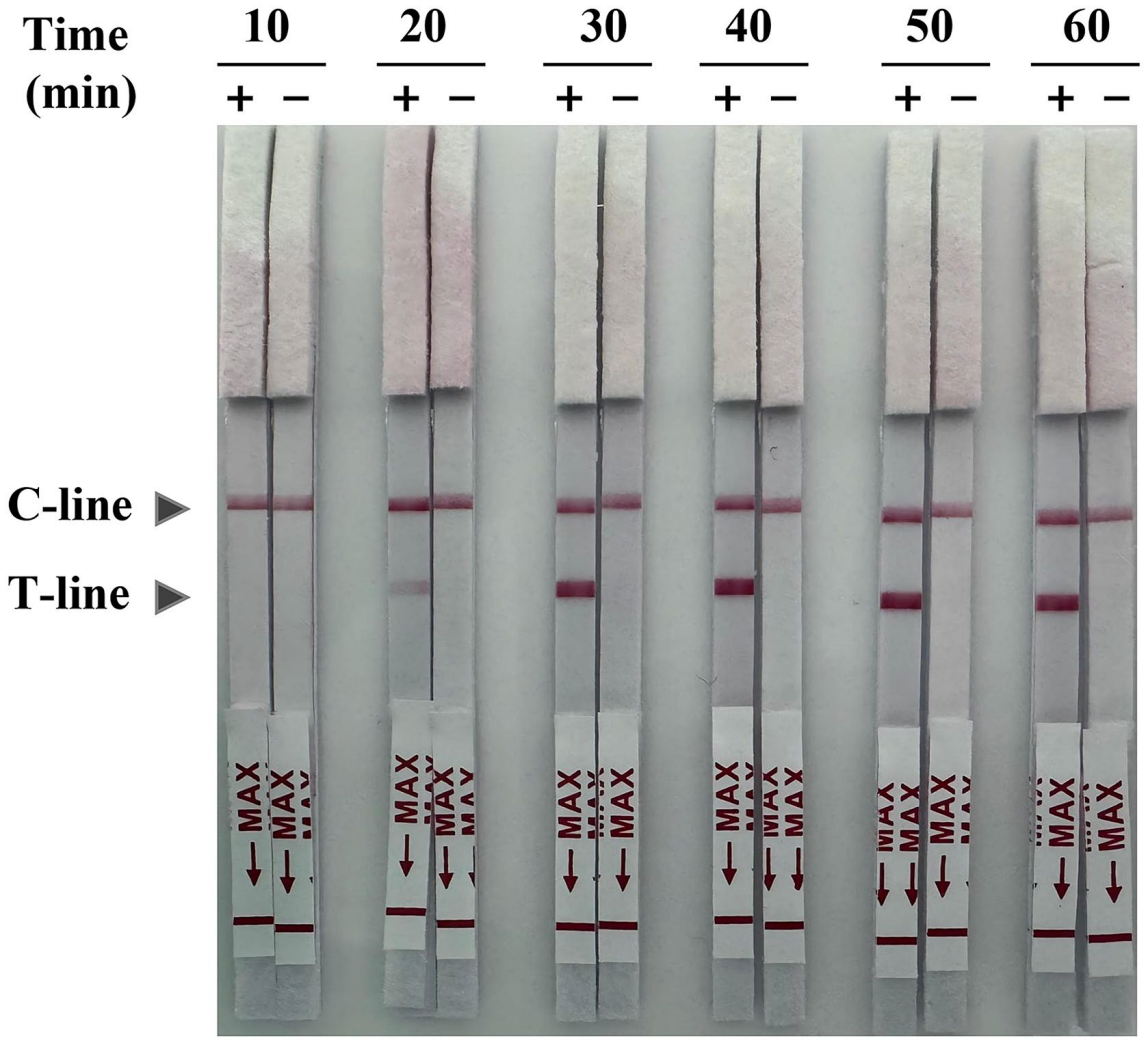
Optimization of the reaction time of LAMP-LFD assay. “+” represent positive reaction with plasmid pMD-TK as a template; “−” and NTC represent negative controls.

The LAMP-LFD assay consists of two core procedures: LAMP amplification and LFD strip detection. Sufficient accumulation of amplicons from the LAMP reaction is required to generate more intense bands on the LFD strip. Accordingly, determining the optimal LAMP reaction duration is critical to the detection performance of LFD. We systematically evaluated the detection efficiency of LFD under different LAMP reaction times. As shown in Figure 4, our experimental results demonstrated that no T-line was observed on the LFD strip after 10 min of LAMP reaction. After 20 min of reaction, a faint T-line appeared on the LFD strip, indicating that amplicons became detectable. The color intensity of the T-line gradually increased with increasing reaction time, and a more distinct red T-line was visible at 40 min. In conjunction with the optimization results of LAMP reaction conditions, which showed that the real-time fluorescent LAMP reaction entered the plateau phase after 35 min, we determined 40 min as the optimal reaction duration for the LAMP assay.

### 3.3 Establishment of FHV-1 LAMP-LFD assay

FHV-1 LAMP-LFD assay was performed in a total volume of 25 μL by adding 2.5 μL of 10 × primer mix (2 μM each of outer primers, 12 μM each of inner primers, and 6 μM each of loop primers), 2.0 μL of template DNA, and 8.0 μL of nuclease-free water into 12.5 μL of LAMP/RT-LAMP 2 × Master Mix. After amplification at 63 °C for 40 min, the reaction products were 40–120 folds diluted. The LFD test strip was then immersed in the diluted solution for 5–10 min, and the results were observed with the naked eye. The positive reaction showed red bands on both the C-line and T-line, while the negative reaction only showed red bands on C-line. The whole detection procedure of FHV-1 LAMP-LFD assay was depicted in Figure 2C.

### 3.4 Specificity of the LAMP-LFD assay

**Figure 5:**
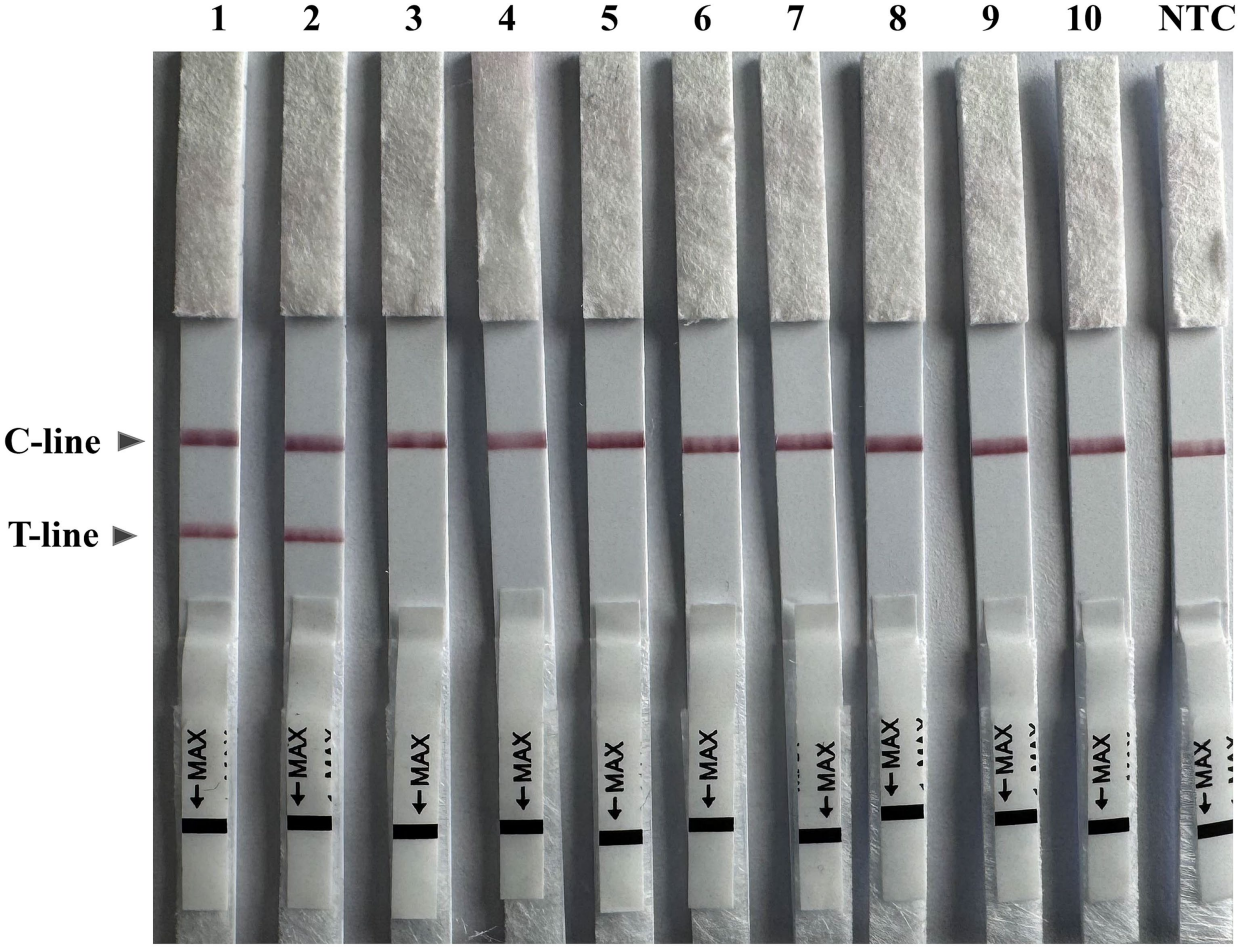
Specificity analysis of FHV-1 LAMP-LFD assay. 1, plasmid pMD-TK; 2, FHV-1 isolate CH-B; 3, FCV; 4, FPV; 5, FCoV; 6, *C. felis*; 7, *M. felis*; 8, *Bordetella bronchiseptica* (*Bb*); 9, *Streptococcus pneumoniae* (*Sp*); 10, CHV-1; NTC, negative control.

As shown in Figure 5, the developed LAMP-LFD assay produced visual red bands at both the T-line and C-line for the plasmid pMD-TK and FHV-1 isolate CH-B. In contrast, other pathogens including FCV, FPV, FCoV, *C. felis*, *M. felis*, *Bb*, *Sp* and CHV-1 only produced visible red bands solely at the C-line, indicating the test results were negative. The specificity analysis demonstrates that the developed FHV-1 LAMP-LFD assay exhibits no cross-reactivity with other common feline pathogens and possesses high specificity.

### 3.5 Comparative sensitivity of the LAMP-LFD assay

**Figure 6:**
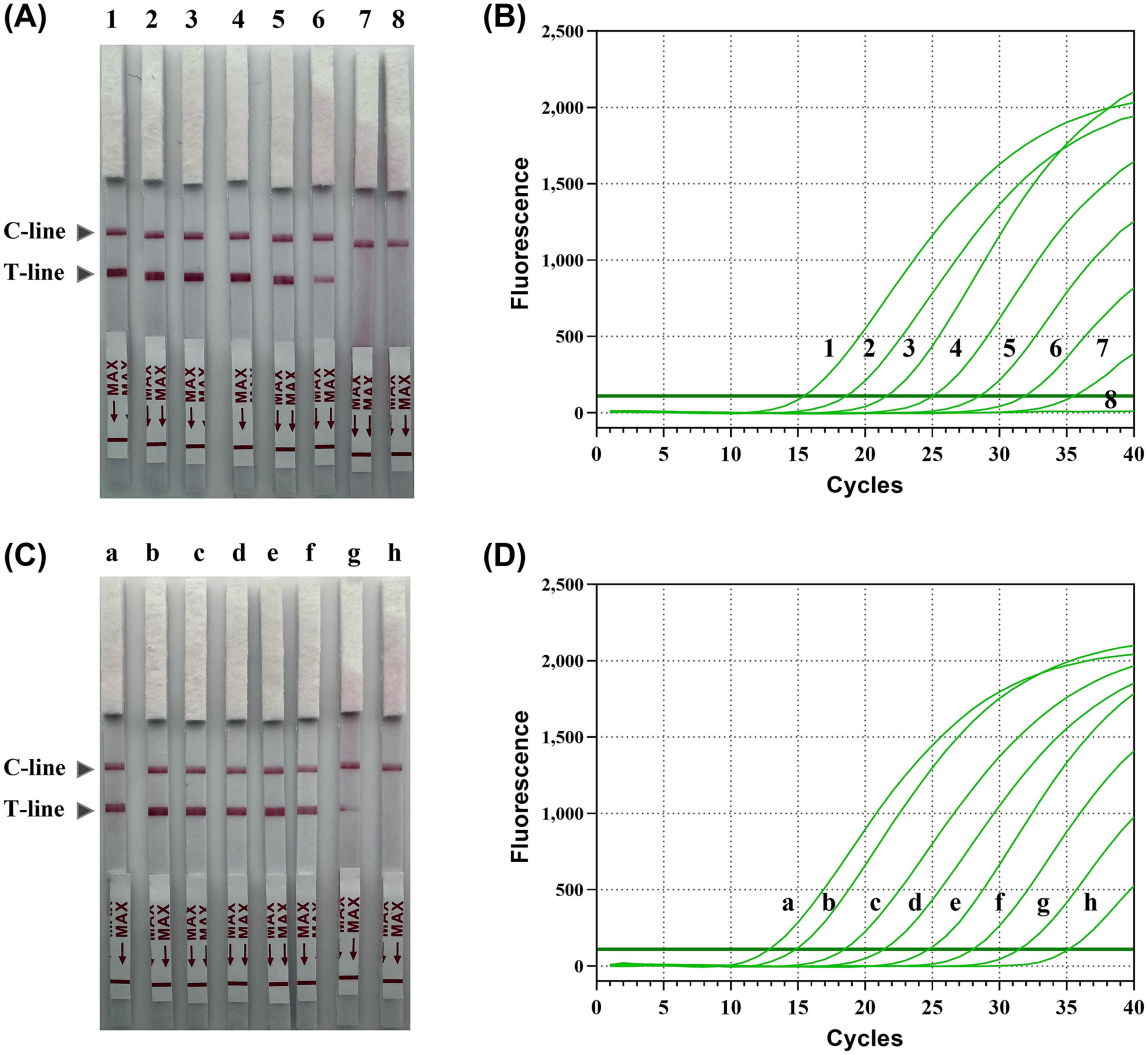
Comparative sensitivity of FHV-1 LAMP-LFD and qPCR assay. The sensitivity analysis of LAMP-LFD **(A)** and qPCR **(B)** assay using pMD-TK plasmid as template. 1–8, pMD-TK plasmid with concentrations ranging from 10^7^ copies/μL to 10^0^ copies/μL. The sensitivity analysis of LAMP-LFD **(C)** and qPCR **(D)** assay using viral DNA from FHV-1 isolate CH-B cultures as template. a–h, FHV-1 isolate CH-B cultures with viral titers ranging from 10^7.5^ TCID_50_/mL to 10^0.5^ TCID_50_/mL.

The limit of detection (LOD) of the FHV-1 LAMP-LFD assay was evaluated using 10-fold serial dilutions of plasmid pMD-TK (ranging from 10^7^ copies/μL to 10^0^ copy/μL) and FHV-1 isolate CH-B cultures (ranging from 10^7.5^ TCID_50_/mL to 10^0.5^ TCID_50_/mL) as templates. The results showed that the LOD of the LAMP-LFD assay was 10^2^ copies/μL for the plasmid pMD-TK and 10^1.5^ TCID_50_/mL for the viral culture (Figures 6A,C). For comparison, qPCR was performed using the same templates, yielding LODs of 10^1^ copies/μL and 10^0.5^ TCID_50_/mL, respectively. The developed LAMP-LFD assay exhibited sensitivity approximately 10-fold lower than that of qPCR (Figures 6B,D).

### 3.6 Repeatability of the LAMP-LFD assay

**Figure 7:**
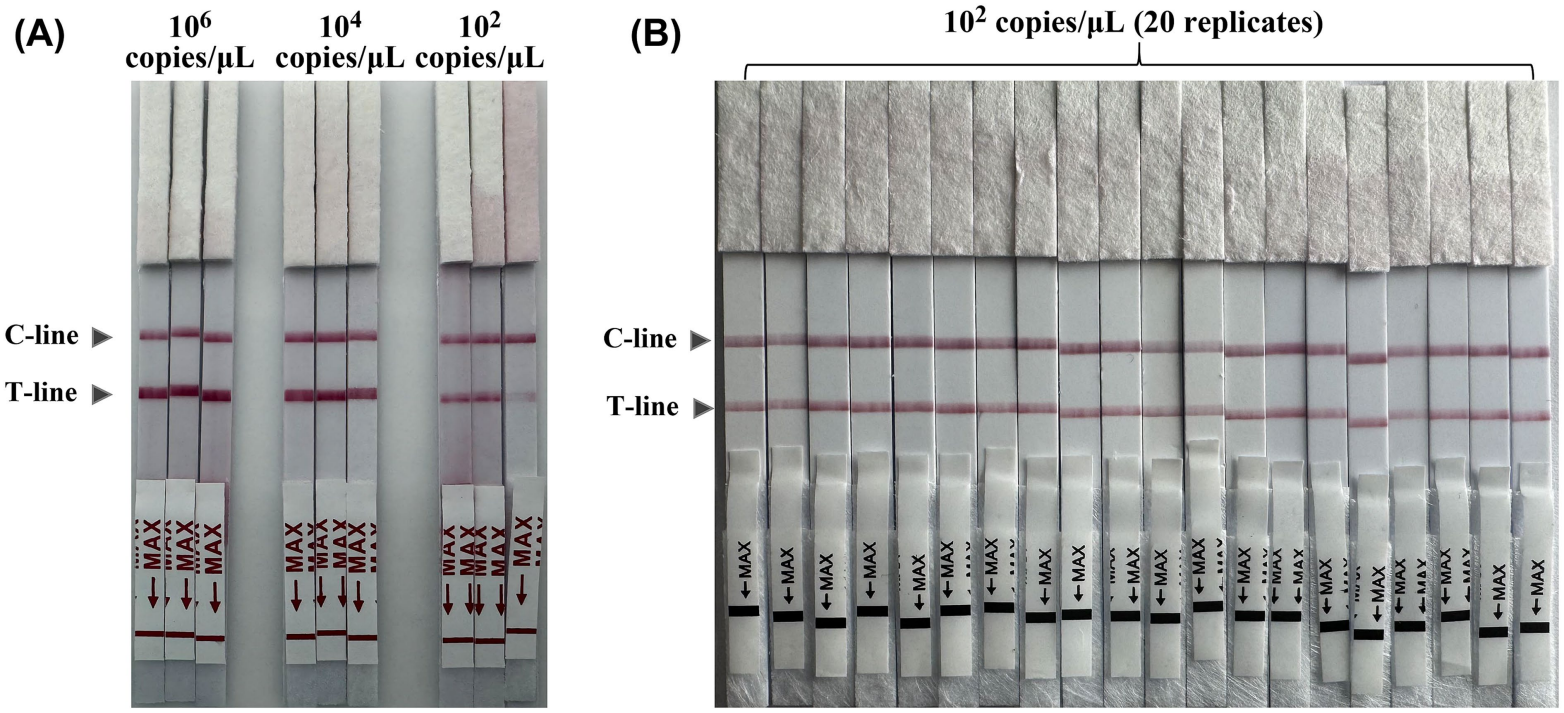
Repeatability analysis of FHV-1 LAMP-LFD assay for strong (10^6^ copies/μL), moderate (10^4^ copies/μL), and weak positive (10^2^ copies/μL) templates. **(A)** Repeatability for plasmids at different concentrations. **(B)** Repeatability for weak positive samples (10^2^ copies/μL).

To evaluate the repeatability of FHV-1 LAMP-LFD assay, plasmids pMD-TK at concentrations of 10^6^, 10^4^, and 10^2^ copies/μL were used as strong positive, moderate positive, and weak positive templates, respectively. In three repeated tests, all positive reactions produced visual red bands at T-line (Figure 7A). For the weak positive (the detection limits of LAMP-LFD assay), a visible but faint T-line could be observed. To further evaluate the detection stability of the LAMP-LFD assay for weak positive samples. A faint T-line was consistently observed across all 20 replicates, yielding a 100% detection rate (Figure 7B). These results indicate that FHV-1 LAMP-LFD assay exhibits good repeatability.

### 3.7 Performance of the LAMP-LFD assay on clinical samples

A total of 87 nasal swabs were detected using FHV-1 LAMP-LFD assay. Compared with the testing results of qPCR assay, the LAMP-LFD detected 43 positive samples, with one sample being negative by qPCR. Among the 44 samples identified as negative by LAMP-LFD, four were positive according to qPCR. Compared to qPCR, the LAMP-LFD assay demonstrated a specificity of 97.56% (with 95% confidence interval (CI): 92.83–100.00%) and a sensitivity of 91.30% (95%CI, 83.16–99.45%), with an overall agreement of 94.25%. The agreement between the LAMP-LFD assay and qPCR was evaluated using Cohen’s kappa statistic, which yielded a kappa (*k*) value of 0.89, indicating almost perfect agreement between the two methods. These results support the clinical utility of the LAMP-LFD assay as a reliable POCT method.

## 4 Discussion

FHV-1 is one of the major pathogens responsible for feline URTD, exhibiting high prevalence rates worldwide—particularly in animal shelters, stray cats, and multi-cat households. Moreover, FHV-1 infection is an important cause of euthanasia for infected cats and can lead to increase higher cost of medical care, and the possibility that affected cats will not be adopted. A retrospective investigation in three diagnostic lab in North America from 2011 to 2020 revealed that *M. felis* (42.2%, 184/436), FCV (31.5%, 119/378), and FHV-1 (23.0%, 94/407) were the most frequently detected pathogens in domestic cats suspected URTD (26). The most common co-infections identified were between *M. felis* and FCV, as well as between *M. felis* and FHV-1. Cavalheiro et al. investigated the morbidity of FHV-1 in domestic cats in Mato Grosso do Sul, Brazil (27). Among 152 tested cases, 84 cats were positive for FHV-1, with a prevalence of 55.26%. In a separate study, Fernández et al. surveyed the morbidity of pathogens caused URTD in cats in Spain and reported an FHV-1 incidence rate of 18.5% (48/260), which was second only to those of FCV (49.6%) and *M. felis* (37.3%) (28). In China, FHV-1 also exhibits a high prevalence among domestic cats. A study involving 400 cats with URTD admitted to animal hospitals across 12 provinces from 2022 to 2023 reported an FHV-1 incidence rate of 21.50% (86/400) (29). Similarly, another study conducted from 2019 to 2022 in 20 animal hospitals in Wuhan city involving 1,158 cats with URTD found an FHV-1 incidence rate of 15.5% (180/1,158) (30). These investigations collectively confirm that FHV-1 remains a significant pathogen seriously threatening the health of domestic cats worldwide. Furthermore, FHV-1 infection occurs frequently in both captive and wild felids. Huang et al. reported a positive rate of 17.3% (56/324) in captive Siberian tigers in northeastern China (15), while Witte et al. documented an incidence rate of 35% (50/144) among cheetah cubs born in six zoos (13). These findings indicate that FHV-1 poses a serious threat to the conservation of wild felids. Hence, there is an urgent need to develop rapid, specific, and sensitive diagnostic methods for FHV-1 to help control its spread.

LAMP first developed by Notomi et al. (31), is an isothermal amplification technique that employs 4–6 primers targeting 6–8 distinct regions of the target gene (24). Using a strand-displacing DNA polymerase, the reaction amplifies the target gene under constant temperature, achieving exponential amplification within 15–60 min. The amplification products can be detected through various methods, including agarose gel electrophoresis, turbidimetry, colorimetry, fluorescent amplification curve, as well as lateral flow dipsticks (LFD) (24, 25). Currently, LAMP technique has been widely adopted for pathogens detection. The selection of target genes is critical to the specificity and efficiency of LAMP. The genome of FHV-1 is highly conserved, with only one known serotype and genotype, and exhibits extremely high sequence identity among different strains. TK gene, a highly conserved region of FHV-1, has been frequently utilized in the development of conventional PCR, nested PCR, TaqMan qPCR, nanoPCR, and RPA assays (17–21, 32). Therefore, this study selected the conserved TK gene as the target for designing LAMP primers. Reaction conditions significantly influence the sensitivity of LAMP. In this study, we systematically optimized the reaction parameters of the LAMP assay, including amplification temperature, primers concentration and reaction time, and developed a rapidly visual detection method for FHV-1 based on LAMP combined with lateral flow detection.

Feline URTD can be caused by a variety of pathogens, including FHV-1, FCV, *M. felis*, *C. felis*, *Bb*, and Sp. These pathogens often induce similar clinical signs, and co-infections are frequently observed (1, 6, 10, 28). To improve diagnostic accuracy, detection methods against felien URTD must exhibit high specificity without cross-reactivity with other common pathogens. In this study, the specificity of the FHV-1 LAMP-LFD assay was evaluated using the common URTD pathogens. The results demonstrated that the assay is highly specific, detecting only FHV-1 and showing no cross-reactivity with other related pathogens, confirming its utility in the specific diagnosis of FHV-1 infection. Furthermore, the sensitivity of the LAMP-LFD assay was assessed, revealing detection limits of 10^2^ copies/μL and 10^1.5^ TCID_50_. Although the sensitivity is 10-fold lower than that of qPCR, the method offers advantages such as low cost, independence from specialized equipment, and short testing time, making it more suitable for POCT compared to qPCR.

To further evaluate the clinical utility of the LAMP-LFD assay, its performance was validated using clinical samples. Among 87 nasal swabs collected from cats with URTD, 43 tested positive by LAMP-LFD, yielding a prevalence rate of 49.43%. All qPCR-positive samples with Ct values<35.00 were also positive by LAMP-LFD, whereas four qPCR-positive samples with Ct > 35.00 were negative using the LAMP-LFD assay. Interestingly, one qPCR-negative sample produced a weak positive result in the LAMP-LFD assay. To ensure accuracy, this sample was tested in triplicate, and consistently showed weak positive in all repeated assays. The exact reason for this discrepancy remains to be further investigated. Comparison of the detection results between LAMP-LFD and qPCR showed an overall concordance rate of 94.25%. The kappa value was 0.89, indicating almost perfect agreement between the two methods. In summary, the developed FHV-1 LAMP-LFD assay demonstrates high sensitivity, excellent specificity, and strong clinical applicability, representing a promising method for the POCT of FHV-1 in primary veterinary hospitals and field settings.

Currently, isothermal amplification techniques such as RPA and enzymatic recombinase amplification (ERA) have been applied to the clinical diagnosis of FHV-1. For instance, Wang et al. developed an exo-RPA assay targeting the TK gene of FHV-1, which enables real-time quantitative detection within 20 min at 39 °C, with a detection limit of 10^2^ copies/reaction (21). Similarly, Liu et al. established a RPA-LFD assay targeting the FHV-1 gD gene (22). This assay utilizes body heat for 20-min amplification and allows visual readout via LFD strips, achieving a detection limit of 10^3^ copies. These assays appear to offer certain advantages over LAMP for POCT of FHV-1, owing to their shorter amplification time and more easily achievable reaction temperatures. However, these methods also present several limitations. Excessively rapid reaction kinetics may pose challenges for practical implementation, particularly for operators with limited professional training. The requirement for multiple enzymes—such as recombinase, polymerase, and single-stranded DNA-binding proteins—further increases the overall assay cost. In contrast, the LAMP assay simplifies the operational procedure through the use of a ready-to-use master mix, requires only a moderate reaction time, and demonstrates sensitivity comparable to that of RPA and ERA. Moreover, it offers improved cost-effectiveness, making it more suitable for broad clinical application.

Aerosol contamination represents the most critical challenge associated with LAMP assay and is a key issue addressed in this study. The LAMP-LFD assay established herein is not a closed-tube system. This assay involves two steps with potential amplicons exposure—dilution of LAMP products and LFD detection—both of which pose a high risk of aerosol contamination. While closed LFD strips could resolve this problem (33, 34), they would increase the detection cost by nearly five-fold. To mitigate contamination risks, we incorporated UDG enzyme and dUTP into the LAMP master mix—a strategy proven to prevent cross-contamination and significantly reduce aerosol-based spread (35, 36). It remains to be further validated whether the addition of UDG/dUTP can eliminate aerosol contamination in the LAMP-LFD assay established in this study. Nonetheless, the complete elimination of aerosol contamination will ultimately depend on the development of economical and practical closed-tube LAMP-LFD detection devices, which represents a key focus of our future work. Furthermore, the LAMP-LFD assay developed herein cannot differentiate wild-type FHV-1 from attenuated live vaccine strains, limiting its application in cats vaccinated with attenuated live vaccine. Stable single nucleotide polymorphism (SNP) loci in the UL28 and UL44 genes, verified between wild-type FHV-1 isolates and the prevalent F2 attenuated vaccine strain (37, 38), serve as ideal targets for strain differentiation. Our future work will focus on establishing high-resolution melting (HRM)-qPCR and MGB probe-based qPCR assays targeting these SNPs for differential diagnosis.

## 5 Conclusion

In conclusion, a rapid and convenient LAMP-LFD assay targeting the TK gene of FHV-1 was successfully developed and validated in this study. This assay exhibits strong specificity, high sensitivity and good reproducibility, and is simple, fast, independent of specialized equipment and easy to perform with results visualized by the naked eye, making it well-suited for point-of-care testing of FHV-1 in primary veterinary hospitals and field setting. This study provides a novel method for the clinical diagnosis of FHV-1 infection in domestic cats and wild felids, demonstrating considerable potential for clinical application.

## Data Availability

The original contributions presented in the study are included in the article/Supplementary material, further inquiries can be directed to the corresponding authors.

## Footnotes

1
https://primerexplorer.eiken.co.jp/lampv5e/index.html

2
https://m4.igenetech.com/mpref

## Supplementary material

SUPPLEMENTARY FIGURE 1 Detection results based on the visual LFD strips **(A)** and agarose gel electrophoresis **(B)** after amplification using the designed LAMP primers.

SUPPLEMENTARY FIGURE 2 Electrophoretic analysis of the products from specificity test of the LAMP-LFD assay.

SUPPLEMENTARY FIGURE 3 Electrophoretic analysis of the products from sensitivity test of the LAMP-LFD assay.

SUPPLEMENTARY FIGURE 4 Electrophoretic analysis of the products from repeatability test of the LAMP-LFD assay.
