# Supplementary figures and images for: Establishment of a loop-mediated isothermal amplification-lateral flow dipstick assay for the point-of-care testing of feline herpesvirus-1
Source: Front Vet Sci. 2026 Jul 6;13:1863672. doi: 10.3389/fvets.2026.1863672 (PMC13381233; doi:10.3389/fvets.2026.1863672)

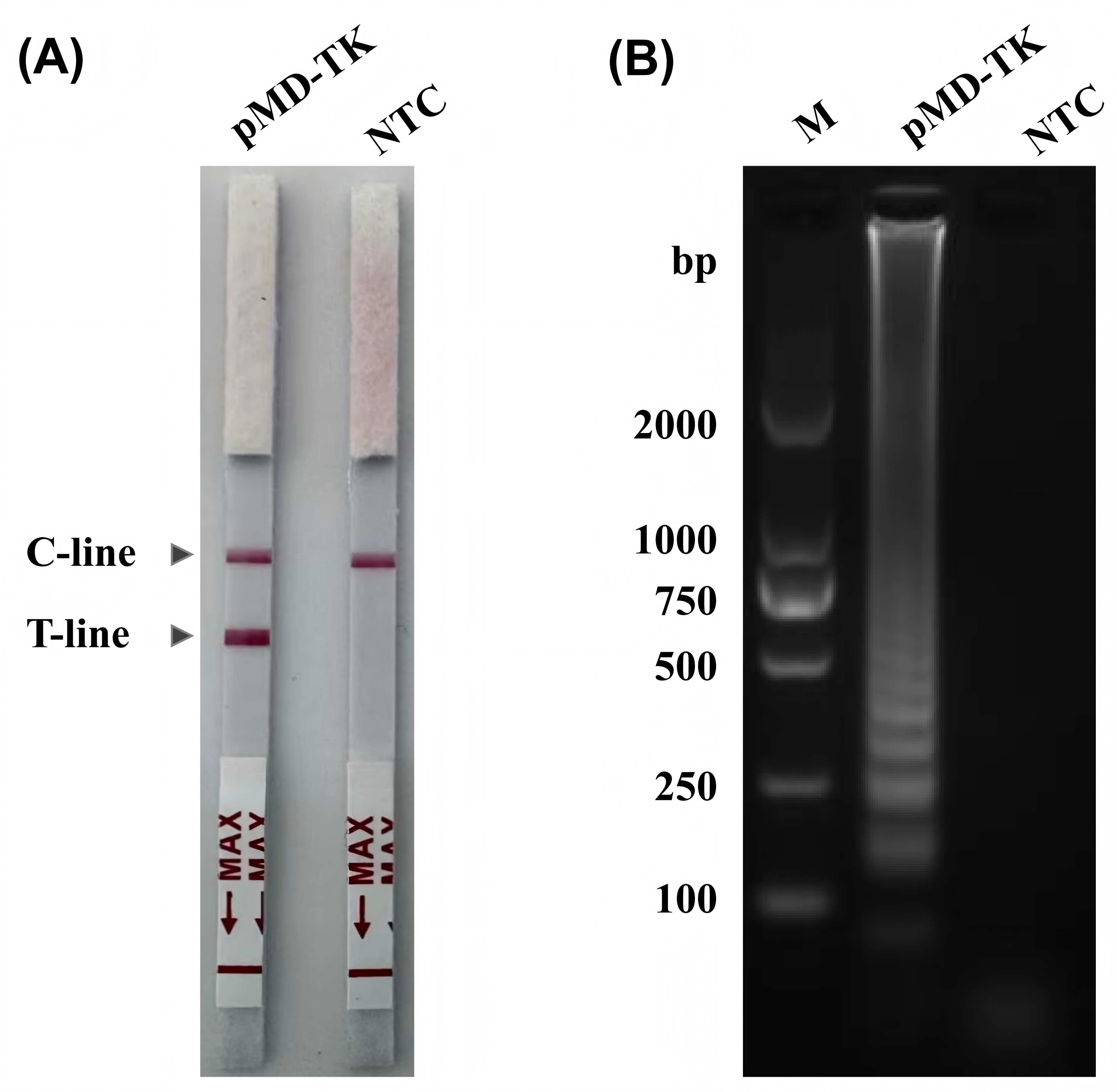

Supplement: SUPPLEMENTARY FIGURE 1 — Detection results based on the visual LFD strips (A) and agarose gel electrophoresis (B) after amplification using the designed LAMP primers. [file Image_1.JPEG]

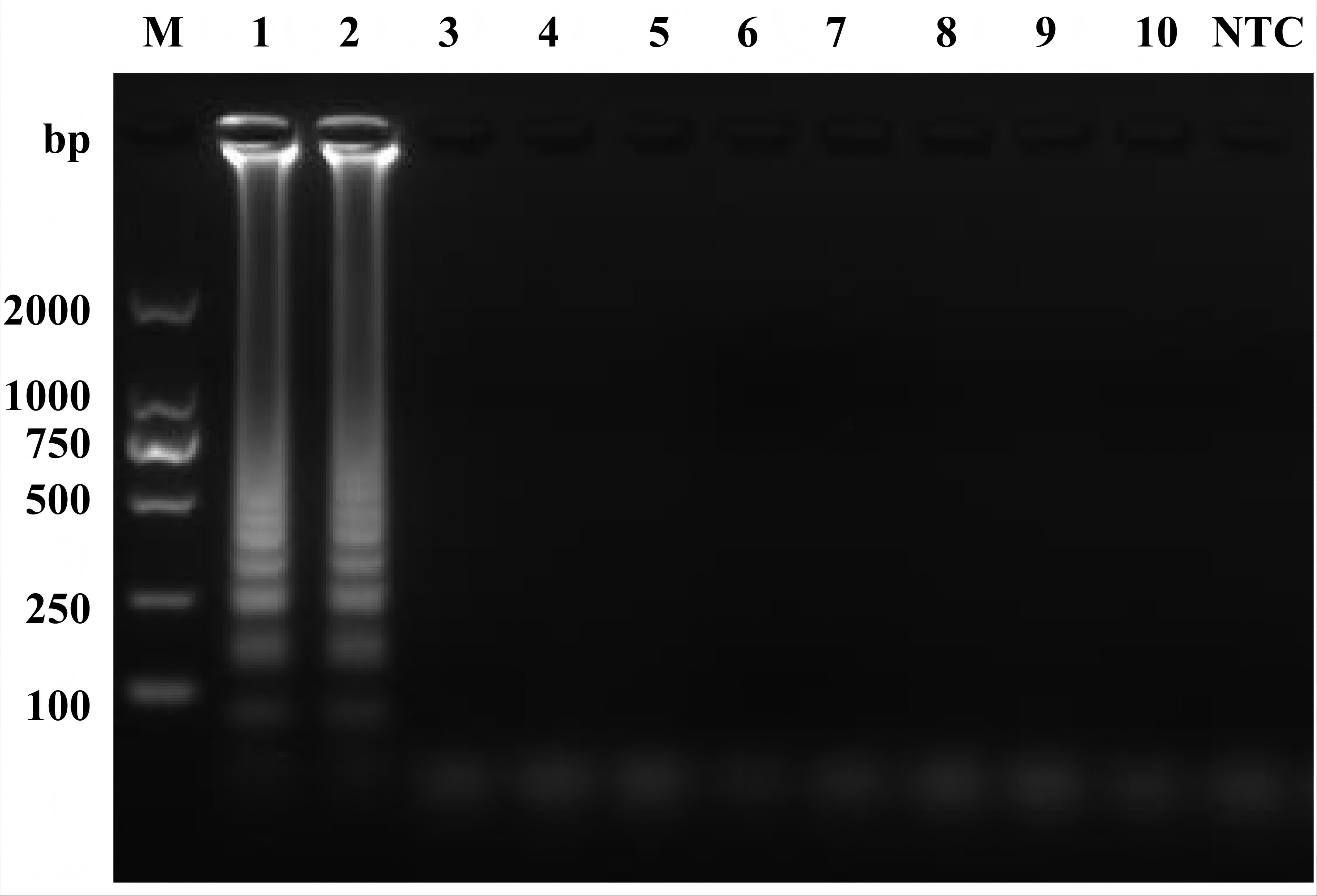

Supplement: SUPPLEMENTARY FIGURE 2 — Electrophoretic analysis of the products from specificity test of the LAMP-LFD assay. [file Image_2.JPEG]

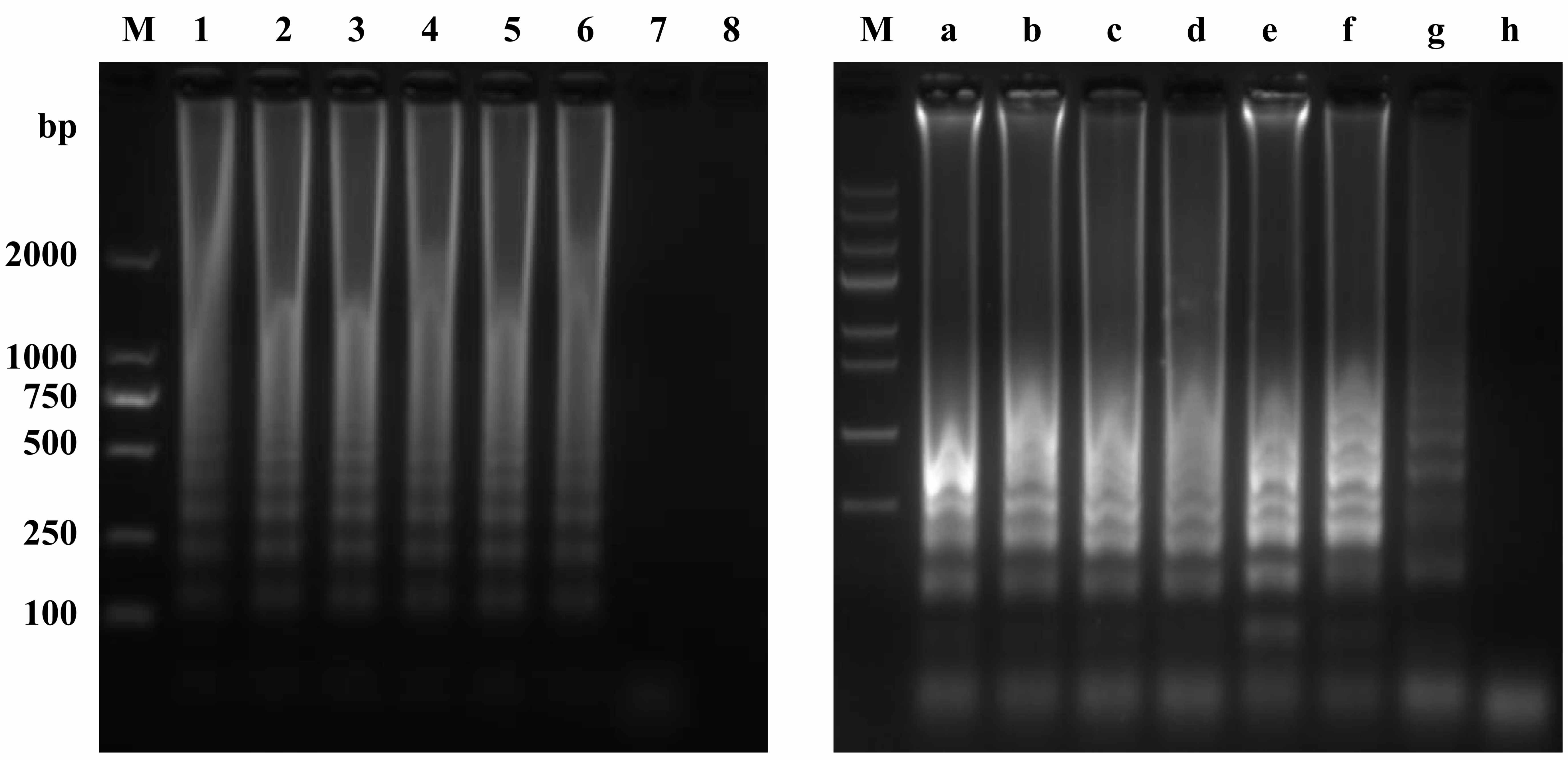

Supplement: SUPPLEMENTARY FIGURE 3 — Electrophoretic analysis of the products from sensitivity test of the LAMP-LFD assay. [file Image_3.JPEG]

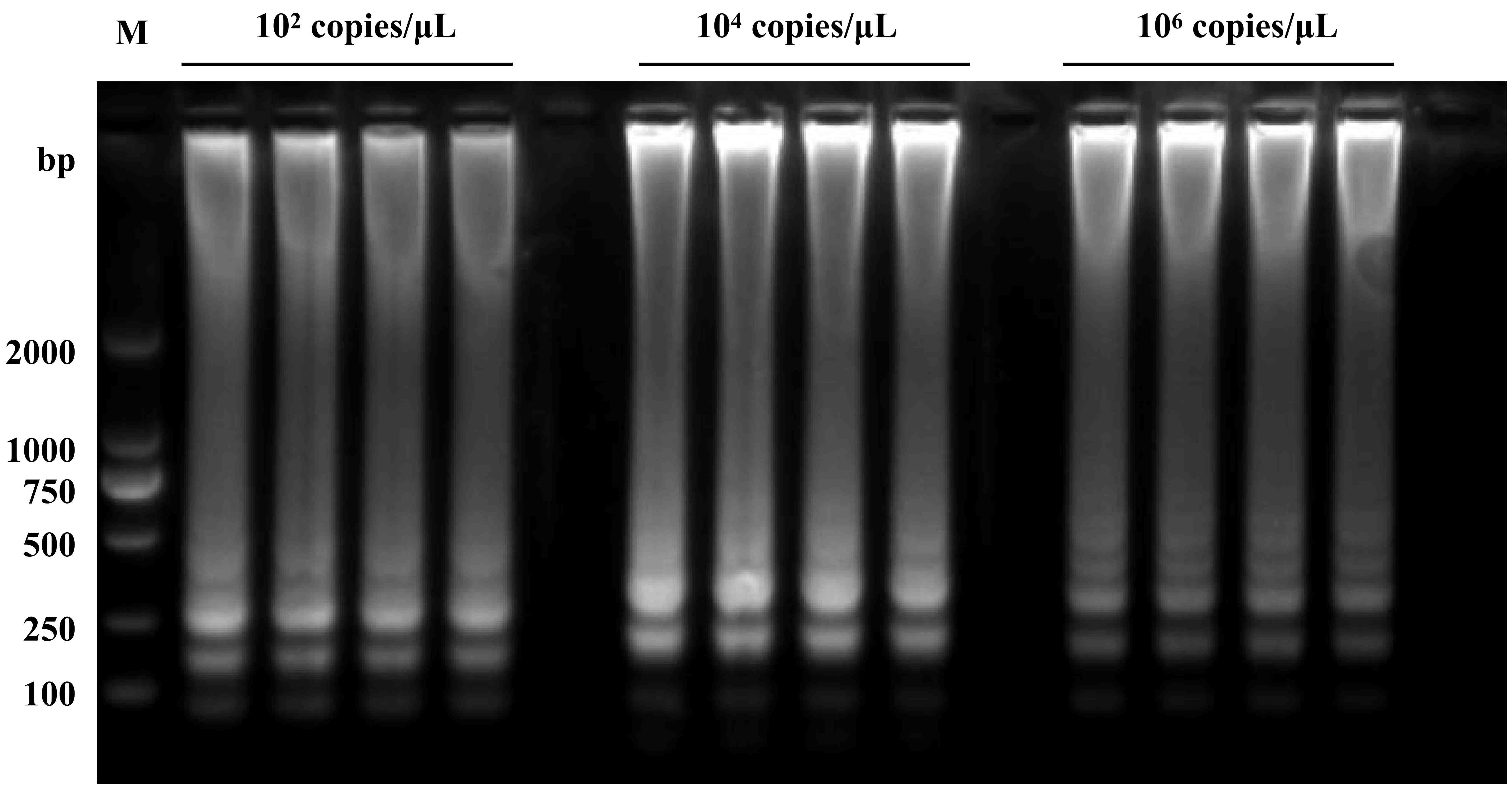

Supplement: SUPPLEMENTARY FIGURE 4 — Electrophoretic analysis of the products from repeatability test of the LAMP-LFD assay. [file Image_4.JPEG]
